# Liposarcome hépatique dédifférencié: à propos d’un cas

**DOI:** 10.11604/pamj.2016.25.119.9494

**Published:** 2016-10-26

**Authors:** Hanane Benzaid, Ahmed Elhankari, Leila Jroundi

**Affiliations:** 1 Service de Radiologie des Urgences, Hôpital IBN Sina, Rabat, Maroc

**Keywords:** Liposarcome, primitif, foie, TDM, IRM, liposarcoma, primitive, liver, Tomodensitometry, MRI

## Abstract

Le liposarcome est une tumeur mésenchymateuse maligne rare, qui siège plus fréquemment au niveau du retro péritoine. La localisation hépatique est extrêmement rare, quelques cas ont été rapportés dans la littérature. Nous présentons le cas d'un un patient de 58 ans, qui se présente pour douleurs abdominales avec à la palpation une masse de hypochondre droit, ayant progressivement augmenté de taille. L'échographie abdominale a montré une masse arrondie, hyper échogène bien limitée. La tomodensitométrie(TDM) abdominale et l'imagerie par résonnance magnétique(IRM) ont confirmé la présence d'une masse qui occupe presque la totalité du foie droit, avec une composante graisseuse. Le patient a bénéficié d'une hépatectomie droite et l'analyse histologique a montrée qu'il s'agissait d'un liposarcome hépatique primitif dédifférencié.

## Abstract (en)

Liposarcoma is a rare malignant mesenchymal tumor usually located in the retroperitoneal space. Hepatic location is extremely rare, few cases have been reported in the literature. We report the case of a 58 year old patient presenting with abdominal pain and the presence of a palpable mass in the right hypochondrium gradually increased in size. Abdominal ultrasound showed hyperechoic well-defined round mass. Tomodensitometry and abdominal magnetic resonance imaging (MRI) confirmed the presence of a mass with a fatty component occupying almost the entire right liver. The patient underwent right hepatectomy and histological examination showed primary undifferentiated liver liposarcoma.

## Introduction

Le liposarcome est une tumeur maligne mésenchymateuse rare, il siège le plus fréquemment au niveau du retropéritoine et au niveau des parties molles des membres. La localisation primitive hépatique reste très rare voire exceptionnelle, quelques cas en littérature anglaise ont été décrits [1]. Dans la plupart des cas, l'imagerie permet d'orienter le diagnostic en mettant en évidence la composante graisseuse de ce type de sarcome. Nous rapportons un cas rare de liposarcome hépatique primitif dédifférencié.

## Patient et observation

**Figure 1:**
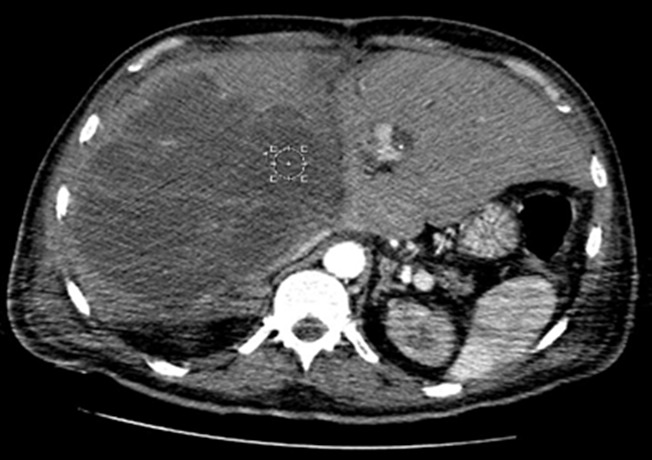
Coupe axiale d’un scanner abdominal passant par le foie après injection du PC

**Figure 2:**
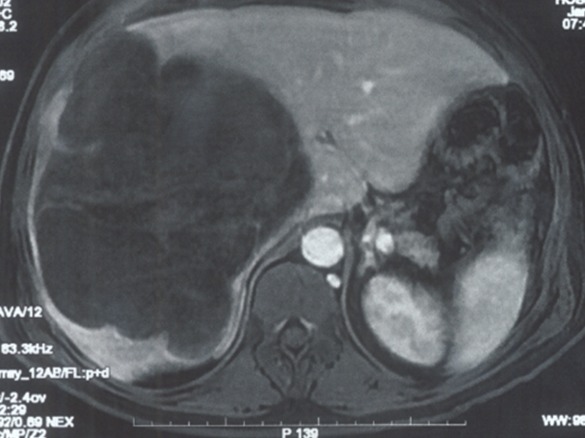
Coupe axiale en pondération T1, en temps artériel après injection du Gadolinium

Il s'agit d'un patient de 58 ans admis aux urgences pour des douleurs abdominales de l'hypochondre doit remontant à trois mois, associées à des frissons et un amaigrissement non chiffré, le tout évoluant dans un contexte d'altération de l'état général. L'examen clinique a trouvé une masse de l'hypochondre droit sans signe d'ictère. Le bilan biologique notamment le bilan hépatique était sans particularité. L'échographie abdominale a révélé une volumineuse lésion hyperéchogène hétérogène, bien limitée, occupant le foie droit. La TDM abdominale avec reconstructions multiplanaires, sans et avec injection du produit de contraste, en temps artériel, temps portal et temps tardif, avait confirmé la présence d'une volumineuse masse occupant presque la totalité du foie droit, n'épargnant qu'une partie du segment IV, mesurant 17 x 14 cm. Cette masse était bien limitée, multi lobulée entourée d'une capsule, avec un contenu de densité graisseuse, cloisonnée, avec rehaussement nodulaires ainsi que des cloisons (Figure 1). Cette masse était à distance des vaisseaux mésentériques. Le reste de l'examen était sans anomalie. Le patient a bénéficié d'une biopsie de la masse hépatique, dont l'étude anatomopathologique montrait une prolifération tumorale d'allure sarcomateuse avec présence de cellules lipoblaste like faisant discuter un liposarcome. L'étude immunohistochimique était en faveur d'un liposarcome dédifférencié. Une IRM corps entier était indiquée à la recherche d'une autre localisation du liposarcome ainsi qu'un bilan lésionnel préopératoire de la masse hépatique et qui montrait un volumineux processus du foie droit, de signal hétérogène en T1, avec des zones en hyper signalT1 hyper signal T2, et d'autres en hypo signal T1, hyper signal T2. Ces dernières se rehaussant de façon hétérogène après injection du Gadolinium, réalisant un aspect de logettes rehaussées en périphérie (Figure 2). Les contours de la masse étaient lobulés, occupant presque tout le foie droit et n'épargnant qu'une partie du segment IV, arrivant à la face antérieure du foie avec effraction capsulaire.les segments I, II et III étaient sans anomalies. La bifurcation portale était englobée. Tronc porte parait libre. Il y avait pas de dilatation des voies biliaires intra hépatiques ni de la voie biliaire principale. Le reste de l'examen à l'étage abdomino-pelvien, cérébral et thoracique étaient sans anomalie, en dehors d'un épanchement pleural droit de moyenne abondance. Le diagnostic de liposarcome hépatique primitif était alors proposé. Le patient avait bénéficié sept jours après d'une ALPPS (Associating Liver Partition and Portal vein ligation for Staged hepatectomy), avec un séjour en réanimation en post opératoires de 10 jours.

## Discussion

Les sarcomes hépatiques sont rares, ils ne représentent que 1 à 2% de toutes les tumeurs malignes primitives du foie [2], prédominés par l'angiosarcome et l'hémangioendothelium. Le liposarcome hépatique reste extrêmement rare, siège habituellement au niveau du retropéritoine et au niveau des parties molles.la localisation hépatique est surtout métastatique. Une dizaine de cas ont été rapporté dans la littérature anglaise. Il s'agit d'une tumeur maligne d'origine mésenchymateuse correspondant à une cellules adipocytaires immatures (lipoblastes). Il existe une prédominance masculine avec un âge moyen de 40 à 60 ans [2], comme notre cas, en dehors du premier cas rapporté en littérature en 1973 d'un liposarcome hépatique chez un jeune de 22 ans [3]. Cliniquement, la plupart des malades sont asymptomatiques. Cependant quand il est volumineux, le liposarcome peut entrainer des douleurs de l'hypochondre droit comme c'est le cas chez notre patient. D'autres symptômes peuvent être retrouvés ; fièvre, nausées, vomissements dans un contexte d'altération de l'état général. Ces signes cliniques sont causés surtout par la compression de structures nerveuses, viscérales ou des voies biliaires. Le bilan biologique reste non spécifique, chez notre patient, le bilan hépatique ainsi que le taux des marqueurs tumoraux étaient normaux. Le diagnostic de liposarcome est habituellement posé grâce aux techniques d´imagerie couramment utilisées. L'échographie reste l'examen de 1ère intention pour l'abdomen, dans la plupart des cas elle montre une masse unique de taille variable avec un aspect échogène et hétérogène, mais ne permet p as de localiser l'épicentre de la masse. À la tomodensitométrie, le liposarcome est hypodense avec des densités graisseuses variant entre -10 et 100 UH [1]. Chez notre patient le scanner a confirmé la présence de la composante graisseuse avec une densité variant entre -20 et -60. Le rehaussement est variable, dépend du degré de différentiation du liposarcome, avec un faible rehaussement dans les formes bien différenciées, avec un rehaussement hétérogène dans la forme myxoïde [4].

Dans notre cas, le rehaussement était mixte et intense vu qu'il s'agissait d'un liposarcome dédifférencié. A l'IRM, le liposarcome présente un hyper signal hétérogène en T1 et T2, s'effaçant en séquence de saturation de la graisse. On peut retrouver au sein de la masse des nodules, septas, des zones d'hémorragie ou de nécrose. Sur le plan histologique, l'organisation mondiale de la santé classe le liposarcome en quatre sous-types: le liposarcome Myxoïde: le type le plus commun dans la population pédiatrique. De haut grade avec risque de métastase; le liposarcome bien différencié: représente 50% des liposarcomes. De bas grade avec risque minime de métastase mais haut risque de récidive local; le liposarcome dédifférencié: siège le plus souvent en rétropéritonéal. Forme de haut grade, renfermant des zones bien différenciées le plus souvent; le liposarcome pléomorphe: forme la plus rare, ne représente que 5 à 10 % des liposarcomes, avec haut risque de récidive locale et de métastase à distance. Les formes myxoïde, dédifférencié et bien différencié au niveau du foie ont été décrites dans la littérature [1, 3, 4]. Notre cas rapporté était un liposarcome dédifférencié avec son aspect biphasique en IRM orientant vers le double contingent graisseux et tissulaire. La biopsie des liposarcomes pose le risque de dissémination. En cas de doute sur la voie d'abord, c'est la bonne coordination entre radiologue et chirurgien qui prime. Le principe habituel est de s'informer sur la voie d'abord chirurgicale afin d'y prasssstiquer la biopsie pour que la cicatrice de biopsie puisse être réséquée lors de l'exérèse de la tumeur. Le point de ponction peut être tatoué à l'encre de chine en fin de procédure pour être retrouvé ultérieurement par le chirurgien. L'échographie ou le scanner sont des techniques d'imagerie adaptées au geste de biopsie à l'aiguille. Le traitement des liposarcomes reste l'exérèse chirurgicale complète avec respect des marges de sécurité, et traitement palliatif si présence de métastase [5]. Notre patient a bénéficié de la technique de ALPPS, il s'agit d'un nouvelle technique d'hépatectomie en 2 temps rapprochés, proposée par une équipe allemande ayant pour acronyme « ALPPS » (Associating Liver Partition and Portal vein ligation for Staged hepatectomy). Cette technique comporte, entre autres, une parenchymotomie hépatique complète séparant le futur foie restant du foie à réséquer. Des taux d'hypertrophie importants sont obtenus, autorisant la résection chez tous les patients. Le bénéfice oncologique reste à confirmer [6]. Le rôle de la radiothérapie n'est pas bien étudié vu la rareté du liposarcome dans la littérature. La chimiothérapie était essayée dans des sous types de haut grade mais sans résultats significatifs. Le pronostic du patient dépend du type histologique du liposarcome, de son grade ainsi que de sa composante nécrotique. La survie à 5 ans chez les patients qui ont bénéficié d'une exérèse complète ou après une radiothérapie est estimée à 50 % [5].

## Conclusion

Le liposarcome primitif du foie est une tumeur exceptionnelle. Compte tenu de leur rareté, la sémiologie de ce type de lésion représente un challenge diagnostique pour les radiologues et la stratégie thérapeutique doit être discutée lors des réunions de concentration pluridisciplinaire afin d'optimiser la prise en charge.
